# Computational Investigation of Structural and Spectroscopic Properties of LOV-Based Proteins with Improved Fluorescence

**DOI:** 10.1021/acs.jpcb.0c10834

**Published:** 2021-02-10

**Authors:** Felipe Cardoso Ramos, Lorenzo Cupellini, Benedetta Mennucci

**Affiliations:** Dipartimento di Chimica e Chimica Industriale, University of Pisa, Via G. Moruzzi 13, Pisa, I-56124, Italy

## Abstract

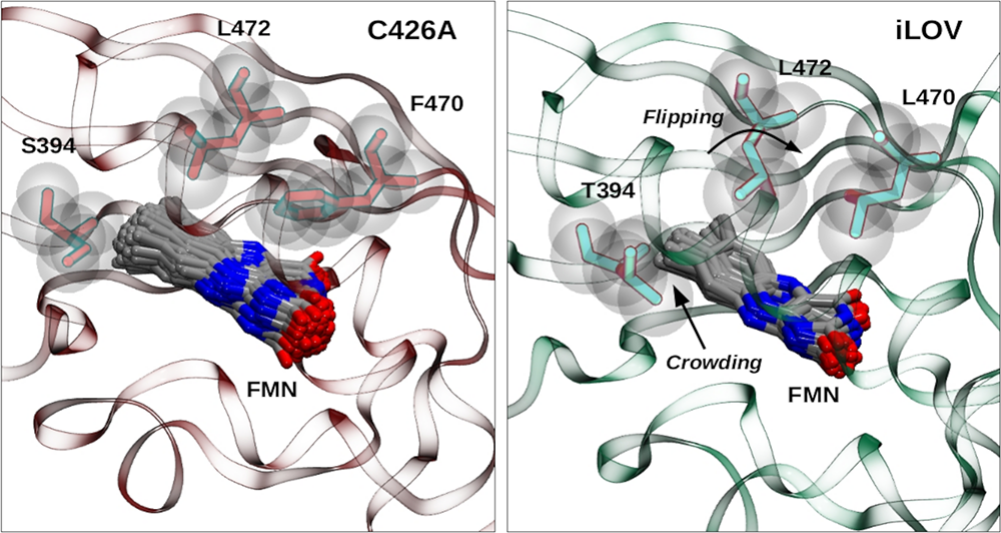

Flavin-based fluorescent proteins are a class of fluorescent reporters derived from light, oxygen, and voltage (LOV) sensing proteins. Through mutagenesis, natural LOV proteins have been engineered to obtain improved fluorescence properties. In this study, we combined extended classical Molecular Dynamics simulations and multiscale Quantum Mechanics/Molecular Mechanics methods to clarify the relationship between structural and dynamic changes induced by specific mutations and the spectroscopic response. To reach this goal we compared two LOV variants, one obtained by the single mutation needed to photochemically inactivate the natural system, and the other (iLOV) obtained through additional mutations and characterized by a significantly improved fluorescence. Our simulations confirmed the “flipping and crowding” effect induced in iLOV by the additional mutations and revealed its mechanism of action. We also showed that these mutations, and the resulting differences in the composition and flexibility of the binding pockets, are not reflected in significant shifts of the excitation and emission energies, in agreement with the similarity of the spectra measured for the two systems. However, a small but consistent reduction was found in the Stokes shift of iLOV, suggesting a reduction of the intermolecular reorganization experienced by the chromophore after excitation, which could slow down its internal conversion to the ground state and improve the fluorescence.

## 1 Introduction

Green fluorescent protein (GFP) has revolutionized the imaging of dynamic processes within living cells.^1^ However, the use of GFP as *in vivo* reporters is limited by some environmental and cellular factors impeding either chromophore formation or fluorescence activity.^2,3^ In this context, the so-called flavin-based fluorescent proteins (FbFPs) emerged as an alternative class of FPs.^4−7^ FbFPs are derived from a highly conserved family of blue light photoreceptors known as light, oxygen, and voltage (LOV) sensing proteins. In nature, LOV proteins typically associate with flavin mononucleotide (FMN) to function as blue-light photoreceptors and regulate a serie of cellular processes in both bacteria (stress response and virulence) and plants (phototaxis).^8,9^

Natural LOV domains bind FMN noncovalently and, upon UVA/blue-light excitation, undergo a reversible photocycle involving the formation of a covalent bond between the chromophore and a conserved cysteine residue with complete loss of fluorescence.^10^ The substitution of the cysteine residue abolishes LOV domain photochemistry and recovers the fluorescence of the bound FMN.^11^ LOV domains photochemically inactivated in this way are inherently fluorescent; however, additional mutagenesis was needed to further improve their fluorescence and photostability.^11−13^ Random and structure-based engineering methods have been combined to generate a large pool of mutants, which are expressed and then selected according to specific properties, such as fluorescence quantum yield, thermal stability, and photobleaching reversibility.^11−15^ In particular, large attention has been given to a specific class of LOV-based reporter variants with improved properties obtained from the LOV2 domain of *Arabidopsis thaliana* phototropin 2 (phot2) through a directed evolution approach based on the DNA shuffling technique and screening toward enhanced fluorescence.^11^

**Figure 1:**
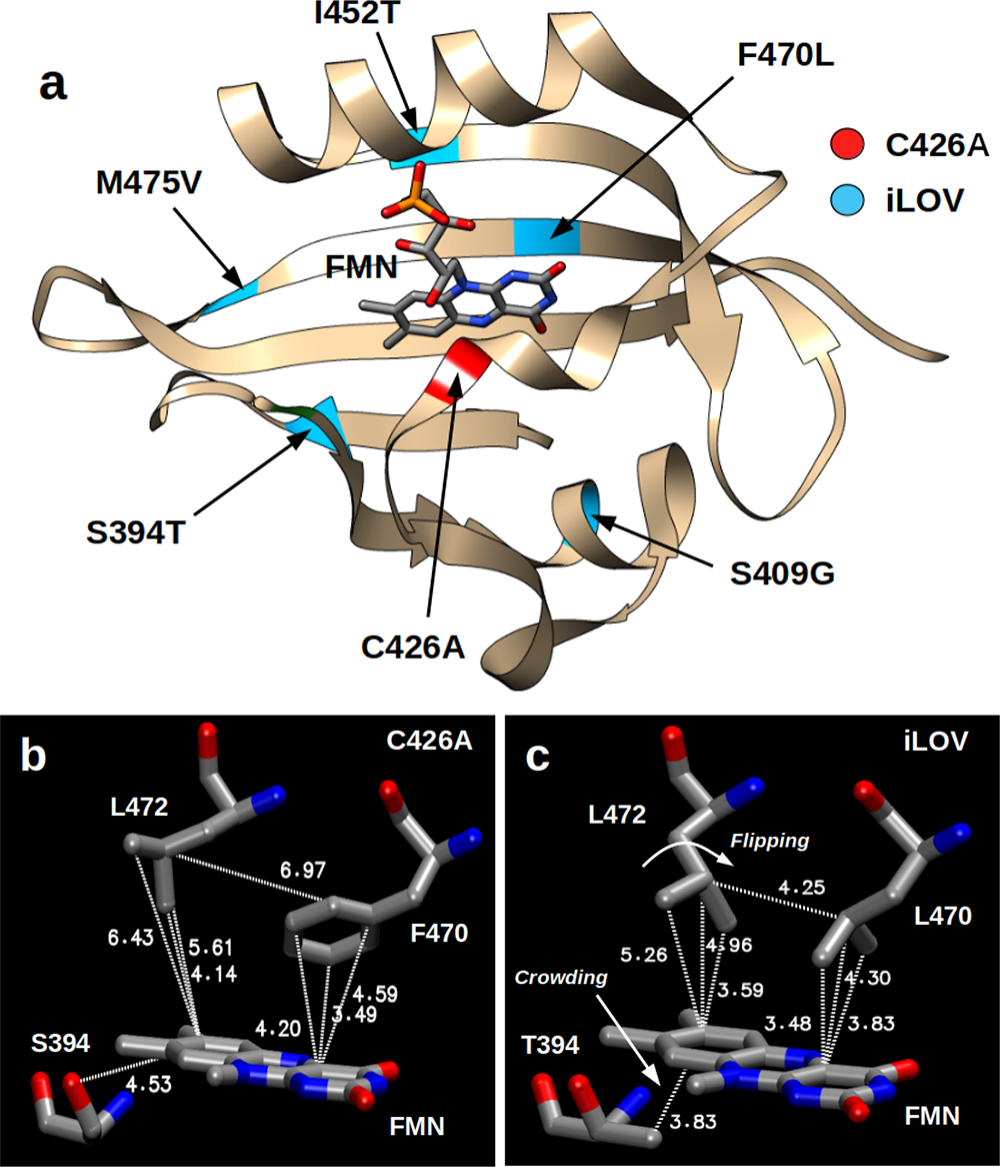
(a) Mutations present in C426A and iLOV with respect to the wild-type LOV2 domain. In the former, the photoactive cysteine was replaced with alanine and in the latter, five additional mutations (S394T, S409G, I452T, F470L, and M475V) were introduced. (b,c) Network of residues involved in the “crowding and flipping” model. The distance values are in angstroms and were obtained from 4EER (C426A) and 4EES (iLOV) crystal structures. Residue numbering is based on the phot2 protein sequence, which is the same found in the PDB structures.

As a first mutation, the photoactive cysteine (Cys426 of *Arabidopsis* phot2) was replaced with alanine to achieve the photochemically inactivated derivative C426A. Five additional mutations (S394T, S409G, I452T, F470L, and M475V, see Figure 1) were introduced to such a derivative leading to an improved LOV (iLOV) showing a substantial increase in fluorescence intensity and emission quantum yield with respect to the single mutant C426A.^11,15^

The iLOV domain was functionally and structurally characterized by Christie et al.^12^ From the crystallographic structures, the authors proposed that the four additional mutations of iLOV stabilize its structure by increasing packing interactions, especially in the flavin-binding cavity. According to this model, the FMN isoalloxazine ring would be rigidified through a “crowding and flipping” process undergone by the T394 and L472 side chains. The crowding is related to the replacement of a serine residue with a threonine residue in the flavin-binding pocket of iLOV (S394T mutation, see Figure 1) while the flipping effect is the rotation of a leucine (L472) side chain in the direction of the chromophore induced by the F470L mutation in iLOV. Both modifications are supposed to increase the stability of flavin in the pocket.

Since all the experimentally obtained FbFPs have similar spectral features (maximum absorption and fluorescence at 447 and 493 nm, respectively),^11,12,15−18^ further optimization efforts have been focused on enhancing the emission color range by introducing single-point mutations in the FMN binding site. The goal in this case is to reach the “biotransparent” window (650–900 nm).^19,20^ Along this research line, a fundamental role has been played by computational studies.^17,21−26^ In particular, these studies have clearly shown that a detailed conformational analysis of the protein is the necessary prerequisite for any research efforts aiming to design variants with further improved fluorescence properties.^26^

Following these findings, here we have performed microsecond-scale simulations of the C426A and iLOV variants originally engineered by Chapman et al.^11^ The goal is 2-fold. First, we aim at characterizing the conformational dynamics of the two systems in solution. Second, we want to verify that the “crowding and flipping” is not an artificial effect due to the crystal structure but it occurs in solution. This goal is achieved through an “in silico” experiment which compares the dynamic interactions between the chromophore and the neighboring residues in the two variants and the resulting absorption spectra and Stokes shifts. The spectroscopic properties are here simulated through a quantum mechanics/molecular mechanics approach which accounts for mutual polarization effects between the QM chromophore and the classical protein residues and solvent molecules.^27^

From this investigation, it comes out that the FMN-protein interactions in the binding pockets are characterized by a large flexibility in the microsecond time scale in both systems. However, this flexibility does not seem to significantly affect the excitation and emission energies. On the other hand, the additional mutations present in iLOV are responsible for a reduced mobility of the isoalloxazine ring of FMN within the pocket. This effect also slightly reduces the extent of intermolecular reorganization experienced by the chromophore after excitation.

## 2 Computational Details

### 2.1 Molecular Dynamics

MD simulations were performed on the two LOV variants described by Christie et al. through crystallography analysis:^12^ the photochemically inactivated LOV2 domain from *A. thaliana*, named C426A mutant (PDB: 4EER), and the iLOV protein containing five single-point mutations (S394T, S409G, C426A, I452T, and F470L) (PDB: 4EES). In Figure S1 of the Supporting Information we report the multiple sequence alignment of wild-type LOV2 domain, C426A, and iLOV.

By using the *tleap* module of AmberTools,^28^ we carried out the preparation of the two investigated systems, their solvation within a truncated octahedron TIP3P water box (with ∼23 000 water molecules), as well as the addition of Na^+^ and Cl^–^ ions at 0.15 M. Extra Na^+^ ions were also add so as to achieve system charge neutrality. The two simulated systems have, in total, approximately 70 000 particles.

The system minimization was done by first minimizing hydrogen atoms, next the solvent components and the protein-chromophore complex, and finally the whole system. For the MD simulations, we first performed the system heating which was divided into two steps: the first one from 0 to 100 K (5 ps in the NVT ensemble) constraining all the system with a harmonic potential (4.0 kcal mol^–1^ Å^–1^) and the second one from 100 to 300 K (100 ps in the NPT ensemble) constraining just the protein backbone. Next, a 5 ns NPT equilibration step at 300 K was done initially applying the same constraint on the protein backbone but releasing the harmonic force constant by 1 kcal mol^–1^ Å^–1^ at each 1 ns. Then, we carried out 5 μs of production at 300 K in the NPT ensemble for both C526A and iLOV systems. Two replica MDs were performed on each system, for a total sampling time of 20 μs.

Both minimization and MD simulations were performed using the Amber16 program employing the *ff14SB*(29) force field for protein. The parameters for the FMN chromophore were obtained from the literature.^30^ In all MD simulations, the time step was set to 2 fs. For system temperature and pressure control we employed a Langevin thermostat and an anisotropic barostat, both implemented in the Amber16. The particle-mesh Ewald algorithm^31^ was used to describe the long-range electrostatic interactions. The MD analysis was performed by using both the *cpptraj*(32) module of AmberTools and locally developed tools.

The network of hydrogen bonds around the FMN chromophore was investigated by computing distances between the FMN and H-bonding residues, as well as among these residues, along both replicas of each system. A principal component analysis (PCA) on these distances, followed by a clustering with the HDBSCAN algorithm,^33,34^ was used to find different conformations of the FMN pocket (clusters). Ten structures were randomly extracted from each cluster to be employed in the following calculations. The interaction of nonpolar residues with the FMN ring was quantified by computing the overlap integral between the pseudoelectronic densities of the FMN ring and the residue side chain:
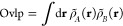
1where the pseudoelectronic densities  (X = *A*,*B*) were computed as a sum of Gaussian distributions centered on the heavy atoms of each fragment:
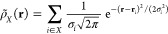
2The standard deviations of the Guassian distributions, σ_*i*_, were taken equal to the van der Waals radii of the elements.

### 2.2 QM/MM(Pol) Calculations

**Figure 2:**
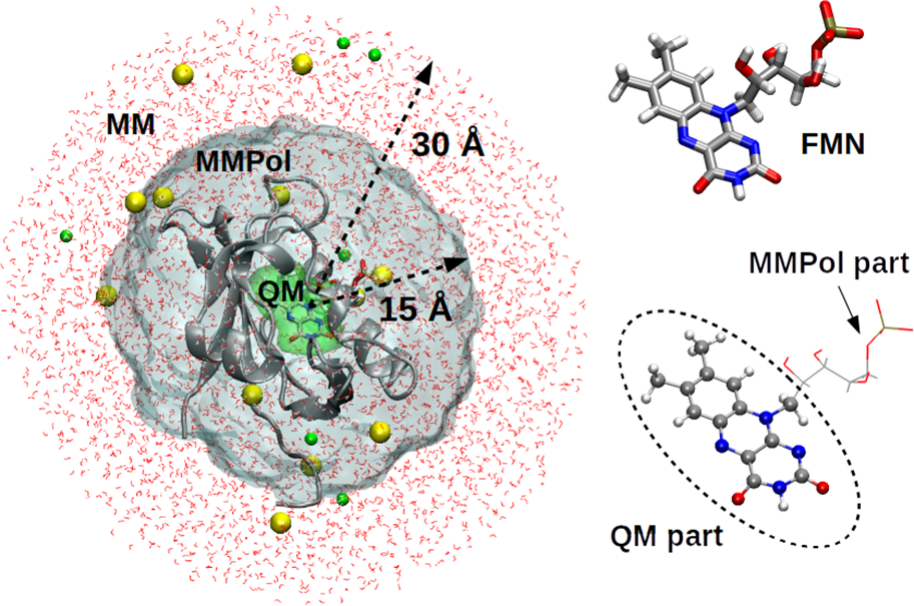
System partitioning employed in the QM/MMPol/MM calculations.

The multiscale calculations, for both crystal structures and MD snapshots, were divided into four steps: (i) ground state (*S*_0_) geometry optimization; (ii) vertical excitation energy calculation; (iii) excited state (*S*_1_) geometry optimization; and (iv) vertical emission energy calculation. The initial coordinates for step i were obtained from the crystal structures and from configurations extracted from the MD trajectories, and the optimized *S*_0_ structures were used as input for step iii. In all steps, only the isoalloxazine group of FMN was included in the QM region while the ribityl tail was treated at classical level (see Figure 2).

All geometry optimizations (steps i and iii) were performed at the ONIOM(QM:MM) level,^35,36^ with the QM subsystem allowed to move and the rest of the system (the ribityl tail of FMN and the protein) kept frozen. For the configurations obtained from MD, a solvation sphere of about 20 Å of radius was defined around the chromophore. The Na^+^ and Cl^–^ ions were stripped from the solvent. The QM part was described at the B3LYP/6-31G(d) level for ground-state optimizations (step i) and TD-ωB97X-D/6-31+G(d) level for the excited state optimizations (step iii). The MM part was described by the same force field used for the MD simulation.

All excitation and emission calculations (steps ii and iv) were performed using a three-layer model (see Figure 2): (1) the QM subsystem described at TD-ωB97X-D/6-31+G(d) level; (2) the protein, the water molecules and ions within 15 Å from FMN described using a polarizable MM model (from now on MMpol), (3) the water molecules and the ions in a shell of 15–30 Å from the chromophore treated at the MM level. The polarizable first shell was described with the *pol12* AL Amber force field.^37,38^

We note that the latter functional gives significantly blue-shifted excitation energies with respect to experiments (about +0.4 eV). A better agreement with experiments was found for B3LYP but, with that functional, the characterization of the excited state was unclear due to artificial mixing. As here the goal was to obtain consistent excitation and emission energies, to predict Stokes shifts, we preferred to use ωB97X-D and obtain well characterized excited states with excitation and emission energies of comparable accuracy, even if both are blue-shifted.

All the calculations have been performed with a locally modified version of Gaussian 16^39^ where the QM/MMpol approach has been implemented.^40,41^

### 2.3 Lineshape Calculations

The homogeneous line shape σ(ω – ω_01_) of the flavin chromophores was computed using the second-order cumulant expansion in the displaced harmonic oscillator (DHO) formalism.^42^
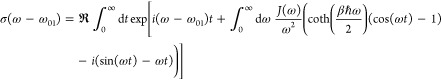
3where the spectral density (SD) *J*(ω) is defined in order to encode the vibronic coupling with all normal modes:
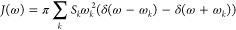
4where *S*_*k*_ is the Huang–Rhys factor along mode *k*. The Huang–Rhys factors were calculated on the crystal structures of both iLOV and C426A, by first computing the ground-state normal modes, in the same ONIOM scheme used for the optimizations in step (i). The excited-state gradient computed at the B3LYP/6-31G(d) level of theory was then projected onto each normal mode to get the Huang–Rhys factor.

The final spectra were computed, for each cluster, by summing the contribution of *N* structures with excitation energies ω_01_^(*j*)^ and transition dipoles **μ**_01_^(*j*)^:
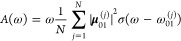
5where σ(ω) is the absorption line shape computed as described above.

## 3 Results and Discussion

### 3.1 Structural Analysis

To achieve a clear description of the structural and dynamic specificities of the two systems which can be related to their different fluorescence behavior, we split the analysis in two parts: one focused on the H-bonding network which characterizes the FMN-binding pocket and the other investigating the specific residues involved in the “crowding and flipping” mechanism, illustrated in Figure 1b,c.

**Figure 3:**
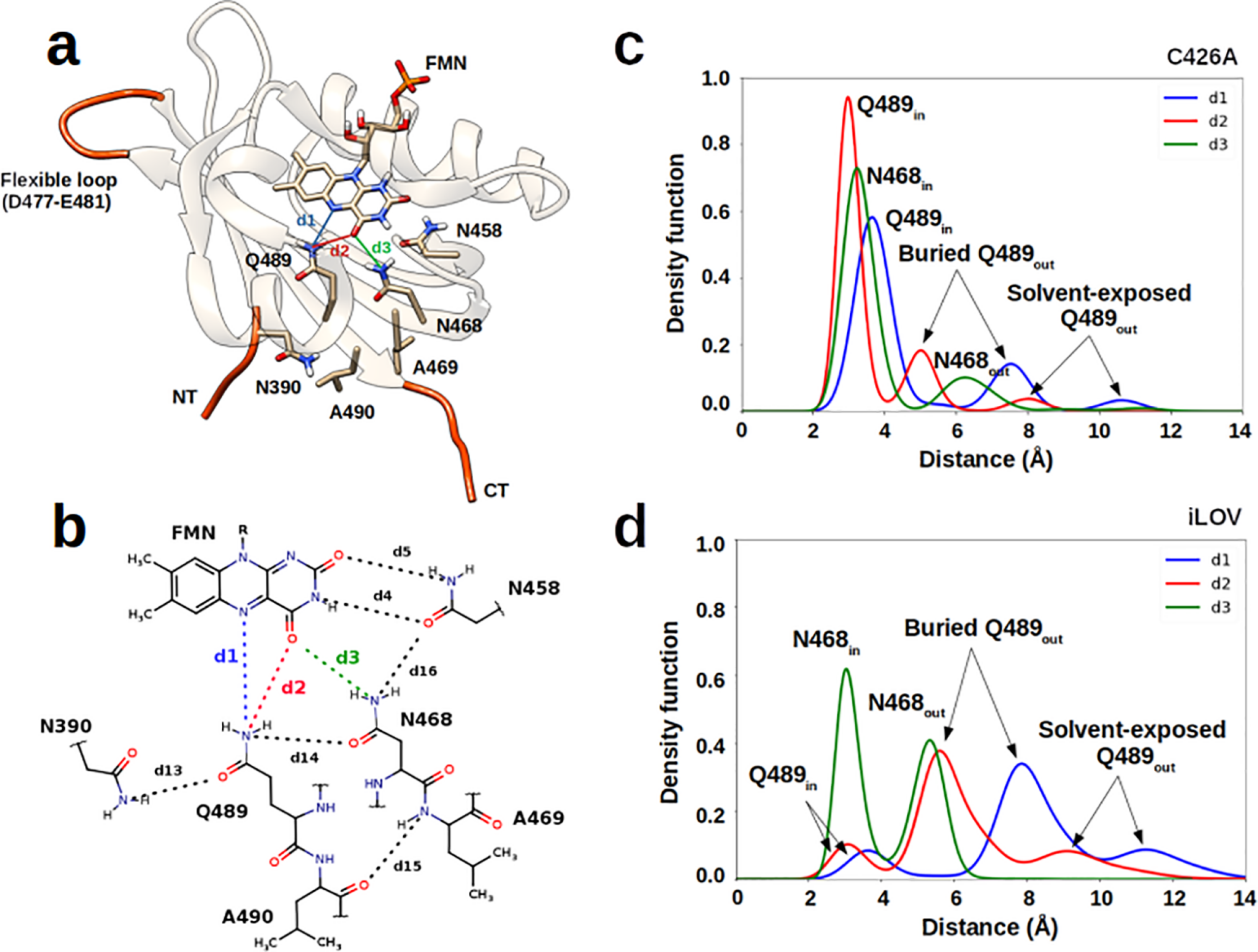
(a) Crystal structure of iLOV showing the conserved flexible residues in the FMN-binding site. In red color are also indicated the regions of the protein that showed great flexibility during our MD simulations (either in C426A or iLOV). NT and CT refer to C- and N-terminal ends. (b) Map of interactions indicating the distances employed in the H-bond analysis (d1–5, d13–16). (c,d) Distributions obtained for FMN-Q489 and FMN-N468 H-bond distances (d1–3) for C426A and iLOV.

As a preliminary and general comment, however, we note that in our microsecond-long MD simulations C- and N- termini showed to be highly flexible in both C426A and iLOV domains, with up to 4 Å RMSD from the crystal structure (Figure S2). Excluding those termini, however, both C426A and iLOV showed a rather rigid protein backbone, with the average RMSD value around 2 Å with respect to the crystallographic structures (Figures S3 and S4). No significant changes in the secondary structure were observed. In one replica, C426A showed increasing RMSD, up to 3 Å after ∼4000 ns. This deviation arises from a conformational change in a flexible loop between residues D477 and E481 (Figure S3). As this loop is external to the protein and far from the binding pocket (Figure 3a), we can exclude an influence of its conformation on the properties of the chromophore.

Moving now to the analysis of the binding pocket, we start by focusing on the extended H-bonding network which is characterized by a total of 22 distances: 12 protein-FMN (d1–12), 8 protein–protein interactions (d13–20) nearby the FMN-binding pocket, and 2 intra-FMN interactions (d21, d22). The full map of the H-bonding distances are reported in Figure S5.

In general, most of the H-bonding interactions here analyzed showed to be stable in both C426A and iLOV during the whole MD simulations. In particular, all the interactions between the ribityl tail of FMN and side chains N425, R427, Q430, and R443 (d7–12) present in the crystal structures were conserved along the MD trajectories. The same occurred for the protein–protein interactions involving the same four residues (d17–20), for the two intra-FMN H-bonds (d21, d22), as well as for the H-bonds between the isoalloxazine ring of FMN and the N458 side chain (d4, d5). On the other hand, differences with respect to the crystal structures have been found for the H-bonding interactions involving residues N390, N458, N468, A469, Q489, and A490 (d1–5, d13–16) (see Figure 3a,b). To give an example of these differences, in Figure 3c,d we compare the distributions of distances between FMN and either glutamine (Q489) or asparagine (N468) residues, in the two systems.

These findings can be explained by noticing that N468 and Q489 chains adopt multiple conformations along the MD trajectories, which correspond to different distances from FMN (Figure 3c,d). As it can be seen from the figures, some conformations are oriented toward the chromophore, with distances less than 4 Å; such conformations are referred to as *in*. Other conformations are instead either solvent-oriented or “buried”, and present distances longer than 5 Å. Such conformations are referred to as *out*. Importantly, we have observed the occurrence of *out* conformations of Q489 after at least 300 ns for iLOV, and after more than 2 μs for C426A (Figures S6, S7). This suggests that the crystal conformation is a local minimum in the free-energy landscape of these systems, which might have been stabilized by crystal packing. The long time scales needed to equilibrate the protein, however, suggest caution in interpreting the relative population of *in* and *out* conformations.

**Figure 4:**
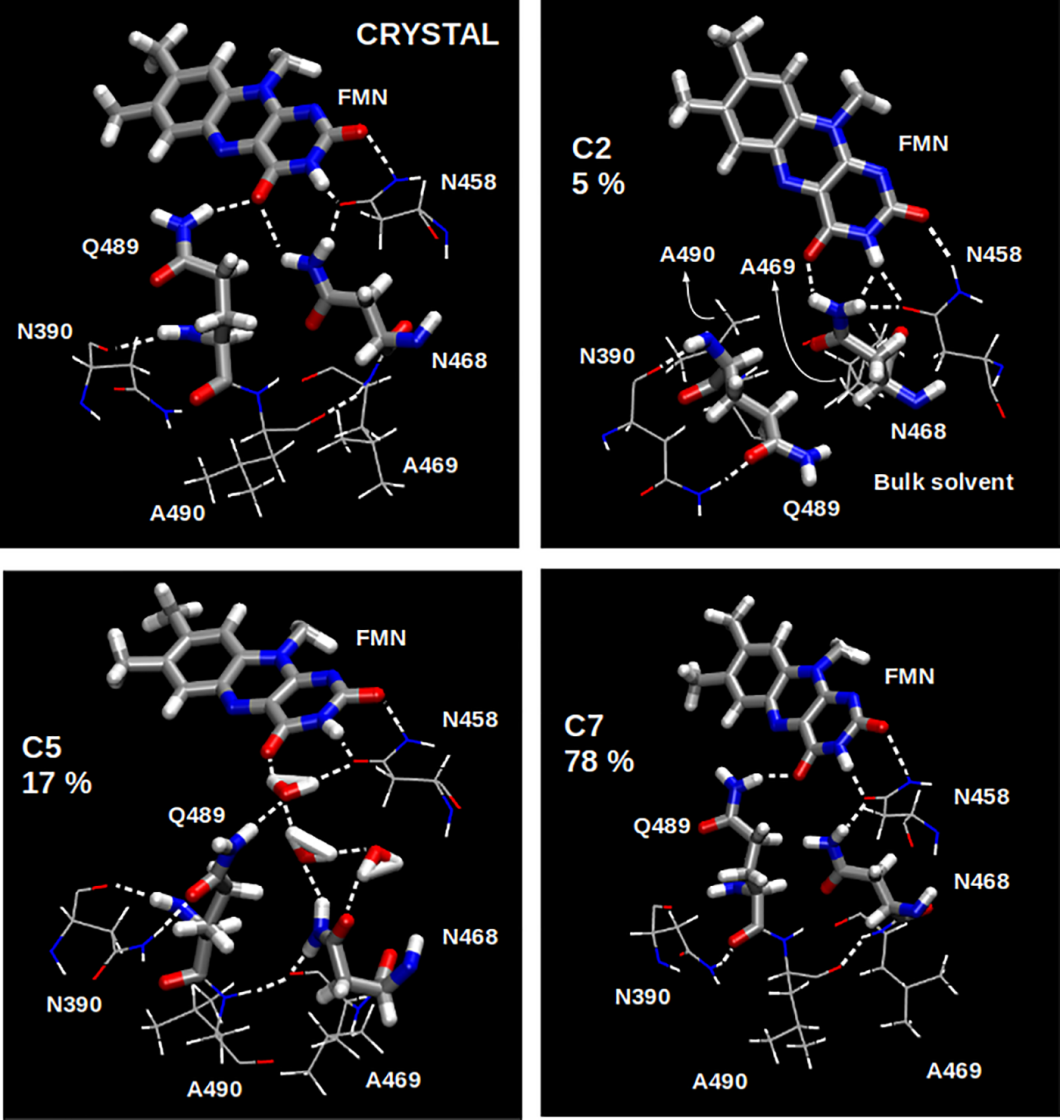
Comparison of the binding pocket in the crystal structure and in representative configurations for the three clusters obtained for C426A (C2, C5, and C7). The percentage values indicate the population of each cluster in the simulated trajectories.

**Figure 5:**
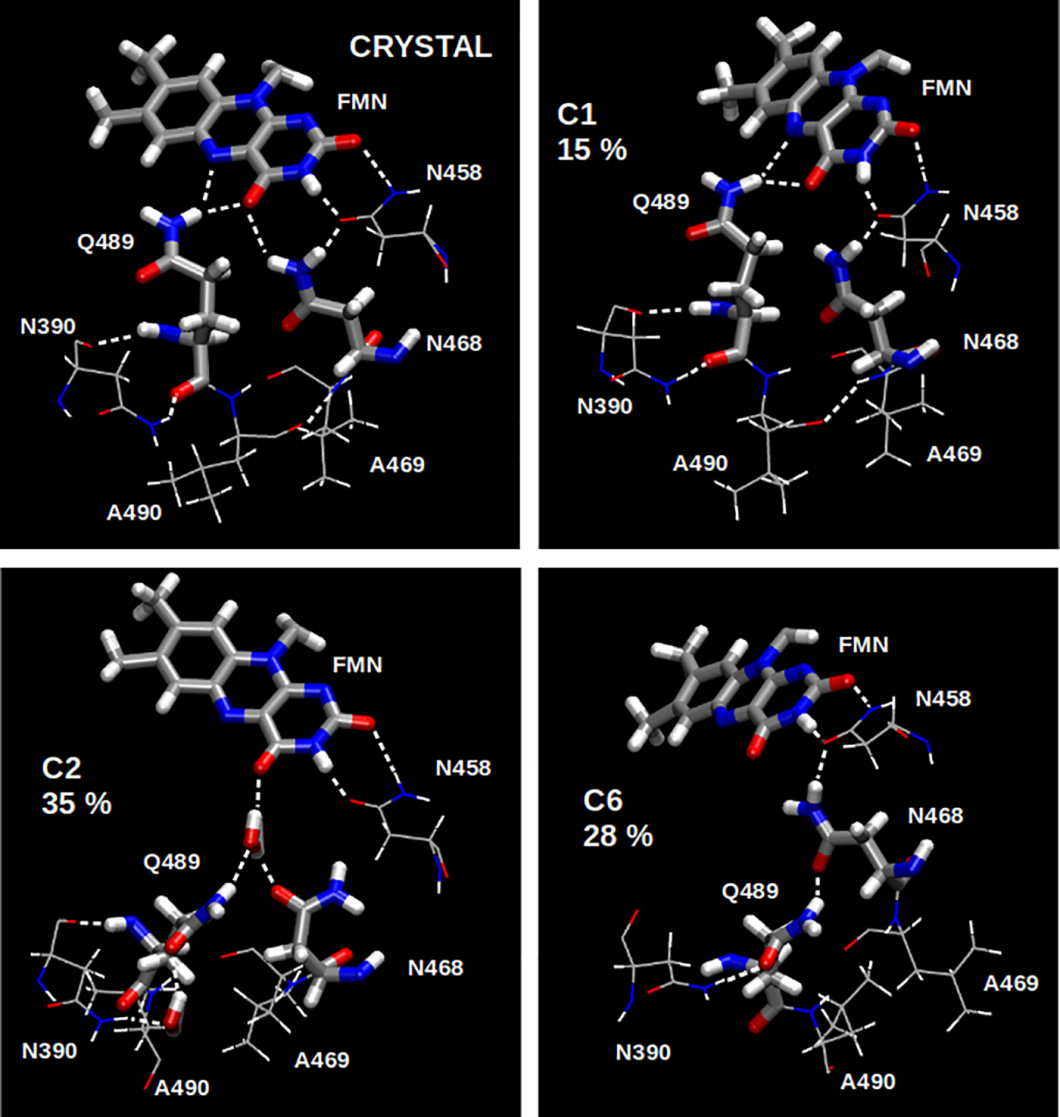
Comparison of the binding pocket in the crystal structure and in representative configurations for the three most populated clusters obtained for iLOV (C1, C2 and C6). The percentage values indicate the population of each cluster in the simulated trajectories.

All these different conformations of the FMN pocket were clusterized on the basis of distances d1–5 and d13–16. The clustering algorithm was able to distinguish Q489_*in*_ and several types of Q489_*out*_ conformations (Figure S6, S7). After excluding clusters with population <1%, we obtained three clusters for C426A (named C2, C5, and C7) and six clusters for iLOV (named C0, C1, C2, C3, C6, and C7). The larger number of iLOV clusters with respect to C426A arises from a greater population of the Q489_*out*_ and N468_*out*_ side chain conformations. A comparison of the network of interactions of FMN with the residues of the binding pocket in the crystal structures and representative configurations for the different clusters are reported in Figure 4 and Figure 5, for C426A and iLOV, respectively. For the latter, only the three most populated clusters are shown while the others are reported in Figure S8.

By analyzing the different clusters we can notice that the Q489_*out*_ and N468_*out*_ orientations favor the entry of water molecules into the active site in both C426A and iLOV. These water molecules can replace Q489 and/or N468 in the H-bonding network with FMN and form a bridge between the FMN and the residues. In such a case, the Q489 side chain adopts a buried conformation Q489_*out*_ relatively closer to the FMN with respect to the solvent-exposed Q489_*out*_ orientation. The two FMN-N458 H-bonds (d4,5), in turn, showed to be quite persistent in both C426A and iLOV (see Figure 4, 5). No *out* orientations were observed for N458.

Here we will use “IN” to refer to the structures in which both Q489_*in*_ and N468_*in*_ occur simultaneously, similarly to what found in the crystal structures. And we will use “OUT” to refer to structures in which either Q489_*out*_ or N468_*out*_ are present. Using this classification, we observe that one cluster of C426A (C7) and one of iLOV (C1) are of type IN. Concerning the OUT structures, our simulations indicate that the Q489_*out*_ side chain can form H-bonds with N390 and N468 (d13 and d14 in Figure S9). Intriguingly, substituting N390 with a serine was shown to improve photostability of the iLOV *in vivo*, probably by restricting the movement of Q489.^12^ In addition, we observed interactions between Q489_*out*_ and water molecules in the active site or from the bulk solvent when Q489 is solvent-exposed (see Figures 4 and 5 and Figure S8). Unlike Q489, N468_*out*_ is never solvent-exposed, but it adopts a buried conformation in both systems. In most of the OUT structures analyzed, the N468_*out*_ conformation is characterized by water molecules bound to the FMN oxygen. Sometimes, the hydrogen bond between FMN and N468 occurs through a bridging water molecule while an interaction between N458 and N468 is established (see C3 in Figure S8).

Previous MD studies of iLOV or natural LOV domains have also reported the change of orientation undergone by the glutamine in the active site.^17,23−25,43^ Experiments and MD simulations showed that K489 is as flexible as glutamine, and prefers an *out* conformation.^17^ MD studies on the LOV1 domain from *Chlamydomonas reinhardtii* also pointed out that the H-bonding interaction between FMN and Q120 side chain (equivalent to Q489 in iLOV) is also unstable, persisting for only 33% of the simulation time (60 ns).^44^ Similarly, Lokhandwala et al.^45^ have observed buried and solvent exposed conformations for the Q204 side chains in MD studies involving a short LOV domain from *Trichoderma reesei*. The N468_*out*_ conformer (or its equivalent in protein sequence) was not reported in previous MD studies, probably due to the short time windows investigated by those simulations (30 to 60 ns). In our simulations, no significant conformational changes of the N468 side chain were observed before 300 ns, suggesting that long simulation times or enhanced sampling methods are needed to assess the conformation of the flavin-binding side chains.

Although largely reported in MD simulation studies, the flexibility of conserved residues in the FMN biding pocket has not been observed in the crystal structures, which consistently present a Q489_*in*_ conformation.^12^ As suggested before,^23^ the more compact Q489_*in*_ conformation might be stabilized by crystal packing. The functional relevance of the dynamics of such residues in natural LOV proteins also remains unclear, but they may have implications for both decay pathways and signal transduction.^44−46^ Regarding the artificial LOV domains, it has been suggested that the Q489 side chain may be involved in both spectral and fluorescence-efficiency tuning.^23^

**Figure 6:**
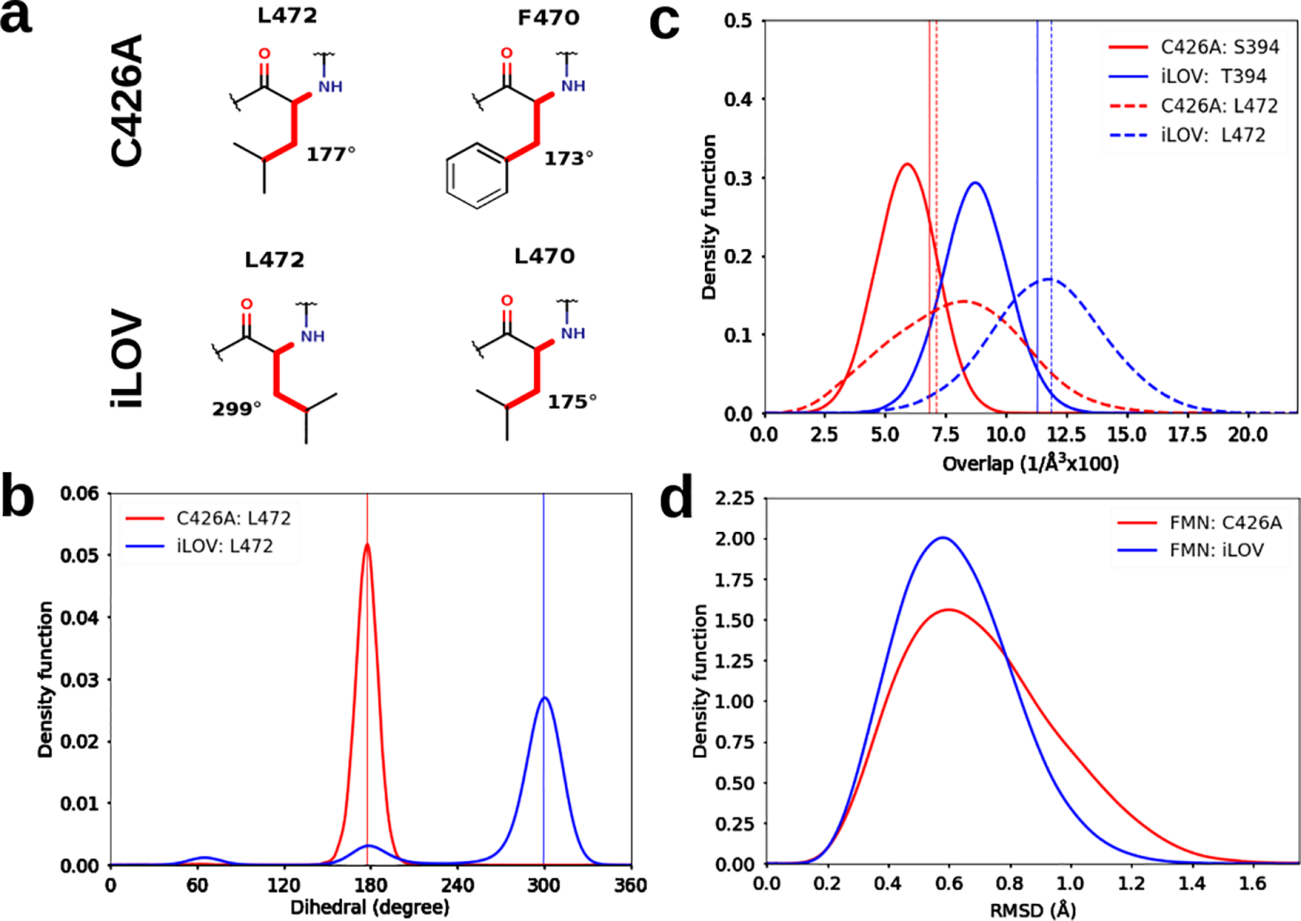
(a) Dihedral angles (red color) analyzed for C426A (top) and iLOV (bottom). The values in degree refer to the crystal structures. (b) L472 dihedral distributions for C426A (red) and iLOV (blue). The vertical lines indicate the values found in the crystal structures. The dihedral distributions for F470 (in C426A) and L470 (in iLOV) are available in Figure S10. (c) Distributions of the overlap between the FMN and the indicated residues for both systems. The vertical lines indicate the overlap values obtained from the crystal structures. (d) RMSD distributions for FMN isoalloxazine ring along DM simulations of C426A (red) and iLOV (blue). The backbone of the protein crystal structures was used as reference for the alignment (the N- and C-terminal residues were excluded).

Instead, Christie et al.^12^ used the evidence given by the crystal structure to propose that the fluorescence increase in iLOV is mainly related to the optimized protein-chromophore van der Waals interactions induced by the “crowding and flipping” mechanism illustrated in Figure 1b,c. To assess if the “flipping” of the side chain of L472, observed in the crystal as a consequence of the F470L mutation, is conserved also in solution, we monitored the dihedral angles of the 470 and 472 apolar side chains present in the active-site of iLOV and C426A (Figure 6). Leucine L472 remains essentially in the crystal conformation, with a rotation of about 120° in iLOV when compared to the single mutant (see Figure 6, top right panel). Also the side chain of residue 470 (Leu in iLOV, Phe in C426A) reproduces the crystal structure geometry for both systems (see Figure S10); however, L470 is somewhat more flexible than F470.

In summary, our MD simulations confirm that the F470L mutation stabilizes the flipping of the L472 residue in iLOV, because L470 has a smaller van der Waals volume compared to F470. This allows the L472 side chain to fit better in the flipped position, closer to the isoalloxazine ring, making this orientation stable also in the presence of thermal fluctuations.

To investigate the crowding effect, instead, we calculated the van der Waals overlap (eq 1) between the FMN and the apolar residues in the positions 394 and 472 of the two systems (namely S394 and L472 for C426A and T494 and L472 for iLOV). The resulting distributions along the MD trajectories are reported in Figure 6c. From these graphs it clearly appears that the FMN in iLOV is significantly more overlapped with both residues than in the other variant. The crowding of the active site in iLOV is induced cooperatively by S394T and F470L mutations, since both side chains of T394 and flipped L472 are closer to the chromophore, thus contributing to a better residue packing around the FMN. Such optimized packing could increase the stiffness of the chromophore in iLOV, as also indicated by the lower RMSD for the isoalloxazine ring obtained from the corresponding trajectories (Figure 6d). All these indications suggest that the presence of T349 and L472 contributes to an improved protein–chromophore interaction which reduces the mobility of the chromophore in the active site of iLOV, exactly as proposed in the crowding mechanism.

### 3.2 Spectroscopic Analysis

The clusters obtained from the analysis of the H-bonding network were finally used in combination with multiscale QM/MMPol calculations, to evaluate the impact of H-bonding dynamics on absorption spectra and Stokes shifts. From each cluster, 10 configurations were extracted and used to compute the chromophore vertical excitation and emission energies (see the Computational Details section and Figure 2).

The calculated vertical energies are reported in Table 1 for each cluster of both systems. In addition, for each system, we also report the population-weighted average values. All clusters give similar results, but some configurations have a more blue-shifted excitation/emission energy. The most blue-shifted cluster presents Q489_*out*_ and N468_*in*_ side chain conformations, with Q489_*out*_ in its solvent-exposed conformation and no water molecules bound to the chromophore (cluster C2 in C426A).

**Table 1:** Calculated Vertical Emission (EMI) and Excitation (EXC) Energies and Stokes Shifts (SS) for the Crystal Structure and the Most Populated Clusters of C426A and iLOV^a^

C426A					
	CRY	C2_OUT_	C5_OUT_	C7_IN_	avg
pop. (%)		5	17	78	
EXC	3.17	3.27	3.23	3.22	3.22
EMI	2.78	2.88	2.84	2.82	2.83
SS	3154	3203	3145	3192	3185

iLOV								
	CRY	C0_OUT_	C1_IN_	C2_OUT_	C3_OUT_	C6_OUT_	C7_OUT_	avg
pop. (%)		11	15	35	7	28	3	
EXC	3.15	3.25	3.21	3.25	3.25	3.25	3.26	3.24
EMI	2.77	2.87	2.82	2.87	2.87	2.86	2.87	2.86
SS	3062	3059	3098	3090	3079	3089	3090	3087

a The weighted average (avg) values are also indicated. For each cluster we also indicate its IN or OUT conformation and the corresponding population (%). EMI and EXC energies are expressed in eV and the Stokes shifts (SS) in cm^–1^.

By comparing the different clusters, we can estimate that the loss of Q489-FMN H-bond causes a blue-shift of about 400 cm^–1^ in the excitation energy. A parallel quantification for the N468-FMN H-bond was not possible because, as mentioned before, in the frames in which we observe the occurrence of N468_*out*_, the N468 side chain is always replaced by water molecules.

**Figure 7:**
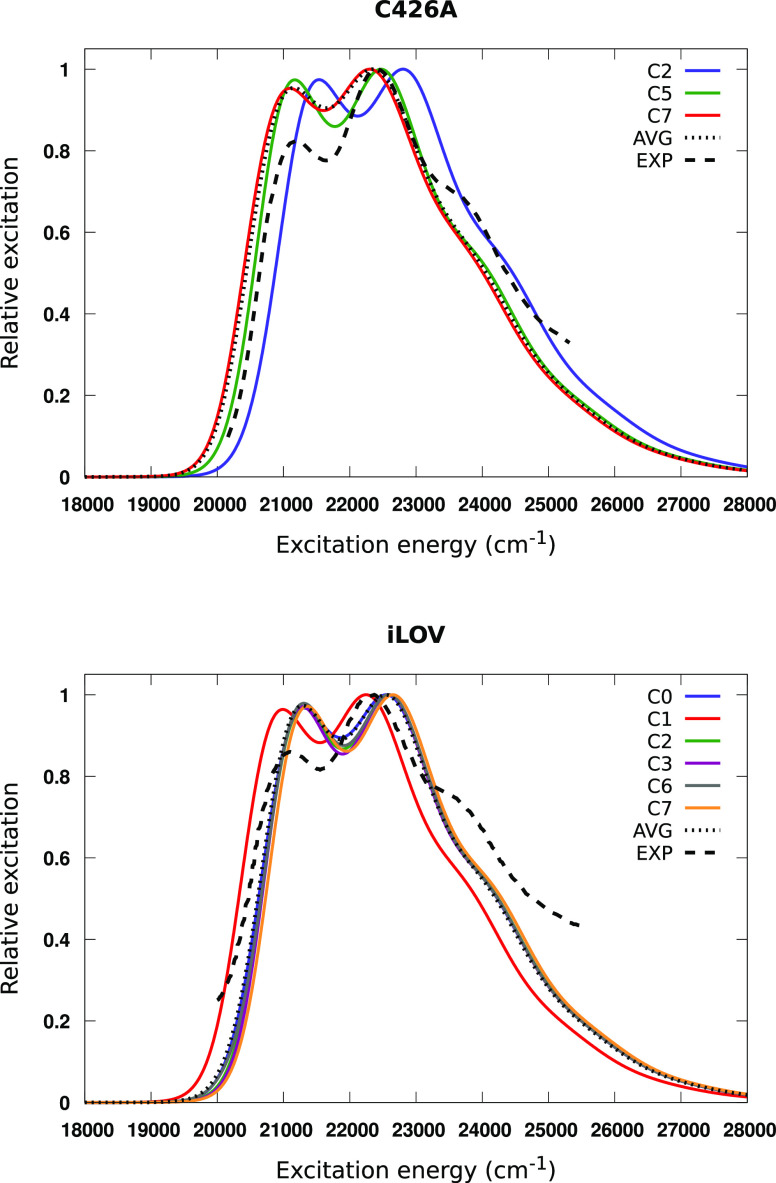
Absorption spectra for C426A (top) and iLOV (bottom) computed from MD using 10 frames for each cluster. The figures show the average spectra for each cluster (solid lines), the weighted average spectra (AVG) for all clusters (dotted line), as well as the experimental absorption spectra (EXP) obtained by Chapman and co-workers^11^ (dashed line). All spectra intensities were normalized so that the maximum is 1. All computed spectra were shifted by −3500 cm^–1^ to match the maximum absorption wavenumber of C426A.

The absorption lineshapes were computed separately for each cluster as detailed in section 2.3. The resulting spectra are reported in Figure 7, along with the population-weighted average and with the experiments. Owing to the large population of in conformations, the calculated average C426A spectrum is essentially the same as the one of cluster C7. On the contrary, in iLOV the average spectrum is determined by the *out* clusters, which are all very similar, and account for ∼85% of the trajectory.

The calculated absorption lineshapes compare well with experiments, reproducing the two main vibronic bands at ∼21 000 and ∼23 000 cm^–1^, as well as the shoulder at higher energies. We can thus conclude that the broadening of the absorption band is mainly vibronic in nature, and that the modulation of pigment–protein interactions has a relatively small effect on the spectrum. While the experimental absorption spectra are very similar for the two proteins, our calculated spectra differ slightly by a small blue-shift for iLOV. We ascribe this difference to the different cluster populations observed in the two sets of MDs. Nonetheless, the spectra of different clusters are shifted by ∼300 cm^–1^, that is, less than the vibronic broadening, suggesting that the IN and OUT conformations could be populated in both C426A and iLOV, without influencing the absorption line shape. Our results suggest that the absorption spectrum of C426A and iLOV is likely the result of several pocket conformations, comprising both *in* and *out* conformations for the glutamine and asparagine side chains.

Regarding the Stokes shift (SS) values (Table 1), no significant differences were observed among the clusters of each system. However, the SS obtained in the C426A clusters is systematically 80–100 cm^–1^ greater than that obtained for iLOV clusters. The Stokes shifts computed on the crystal structures present the same trend for the two systems, with values comparable to the MD ones. From these data, it is clear that the Stokes shift does not depend on the dynamic H-bonding network, but it is apparently controlled by the mutations S349T and F470L discussed above. By hampering the mobility of the isoalloxazine ring, the “crowding” effect slightly reduces the extent of intermolecular reorganization experienced by the chromophore after excitation to the *S_1_* state. We speculate that the same mechanism also contributes to slowing down the internal conversion of FMN toward the ground state. Intriguingly, the measured fluorescence spectra of C426A and iLOV, while very similar,^11^ show some non-negligible differences (Figure S11). Indeed, the fluorescence spectrum of iLOV is narrower and slightly blue-shifted, in agreement with the Stokes shifts calculated in this work.

## 4 Conclusions

We analyzed the microsecond time-scale dynamics of two LOV-based fluorescent protein variants, namely C426A and iLOV, by means of MD simulations. In particular, we characterized the dynamics of the H-bonding network of conserved residues in the FMN binding pocket of the two systems and found a significant flexibility especially for the glutamine (Q489) and asparagine (N486) side chains which undergo conformational changes in time scales in the order of 200–300 ns. These findings stress the importance of a proper sampling for any study aimed at optimizing LOV-derived fluorescent proteins.^26^

Our simulations confirmed the “flipping and crowding” effect induced in iLOV by the additional mutations and revealed its mechanism of action. In particular, the crowding is cooperatively triggered by S394T and F470L mutations. The former leads to a direct packing effect, while the latter allows the conserved L472 side chain to change conformation and fit closer to the chromophore. On the other hand, we showed that these mutations, and the resulting differences in the composition and flexibility of the binding pockets, are not reflected in significant shifts of the excitation and emission energies, in agreement with the similarity of the spectra measured for the two systems. However, a small but consistent reduction was found in the Stokes shift, moving from C426A to iLOV. This suggests that the reduction induced by F470 and S394T mutations in the mobility of the isoalloxazine ring of FMN in iLOV also reduces the intermolecular reorganization experienced by the chromophore after excitation. Here, we hypothesize that the same mechanism could also contribute to slowing down the internal conversion of FMN toward the ground state and to improve the fluorescence of iLOV.

## Supporting Information Available

RMSD plots for the different replicas of the systems. Additional analyses of the H-bonding distances in the binding pockets. Representative structures of the less populated clusters of iLOV (PDF)
