# Supplementary material for: Computational Investigation of Structural and Spectroscopic Properties of LOV-Based Proteins with Improved Fluorescence
Source: J Phys Chem B. 2021 Feb 10;125(7):1768–77. doi: 10.1021/acs.jpcb.0c10834 (PMC7917436; doi:10.1021/acs.jpcb.0c10834)
Supplement: Supplementary file 1 — jp0c10834_si_001.pdf [file jp0c10834_si_001.pdf]

Supporting Information
A Computational Investigation of Structural and Spectroscopic Properties of LOV-based Proteins with Improved Fluorescence

Felipe Cardoso Ramos,* Lorenzo Cupellini, and Benedetta Mennucci*

Dipartimento di Chimica e Chimica Industriale, University of Pisa, Via G. Moruzzi 13, I-56124 Pisa, Italy

E-mail: cfelip2@gmail.com; benedetta.mennucci@unipi.it

```
WT        IEKNFVISDPRLPDNPIIFASDSFLELTEYSREEILGRNCRFLQGPETDQATVQKIRDAI 60
C426A     IEKNFVISDPRLPDNPIIFASDSFLELTEYSREEILGRNARFLQGPETDQATVQKIRDAI 60
iLOV      IEKNFVITDPRLPDNPIIFASDGFLELTEYSREEILGRNARFLQGPETDQATVQKIRDAI 60
          *******:**************.****************.********************

WT        RDQREITVQLINYTKSGKKFWNLFHLQPMRDQKGELQYFIGVQLDGSDHV   110
C426A     RDQREITVQLINYTKSGKKFWNLFHLQPMRDQKGELQYFIGVQLDGSDHV   110
iLOV      RDQRETTVQLINYTKSGKKFWNLLHLQPVRDQKGELQYFIGVQLDGSDHV   110
          ***** *****************:****:*********************
```

Figure S1: Multiple alignment between the protein sequences of the wild-type LOV2 domain (WT), C426A and iLOV. For clarity, residues are numbered according to its position in the simulated protein sequence (considering I387 as the first residue), the same sequence synthesized and expressed by Christie and coworkers.

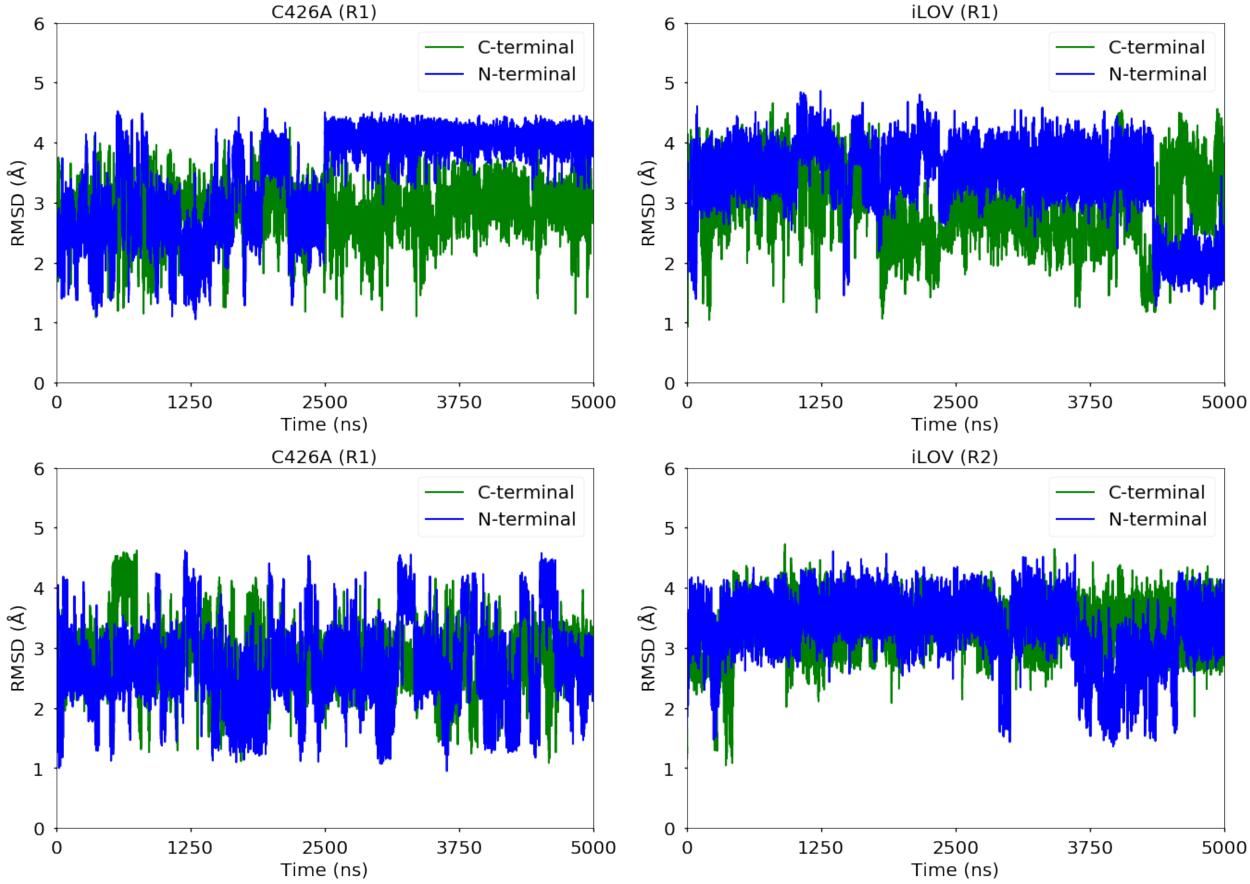

Figure S2: RMSD plots for the C-terminal (residues 1-4) and N-terminal (residues 106-110) extremities of C426A (right) and iLOV (left). R1 and R2 indicates the two MD replicas performed for both systems. This analysis was performed by using the protein crystal structures as reference for the structural fitting. The N-terminal (residues 1-4) and C-terminal (residues 106-110) ends, as well as the flexible loop (residues 91-95), were not considered for the fitting. The hydrogen atoms were excluded from the analysis.

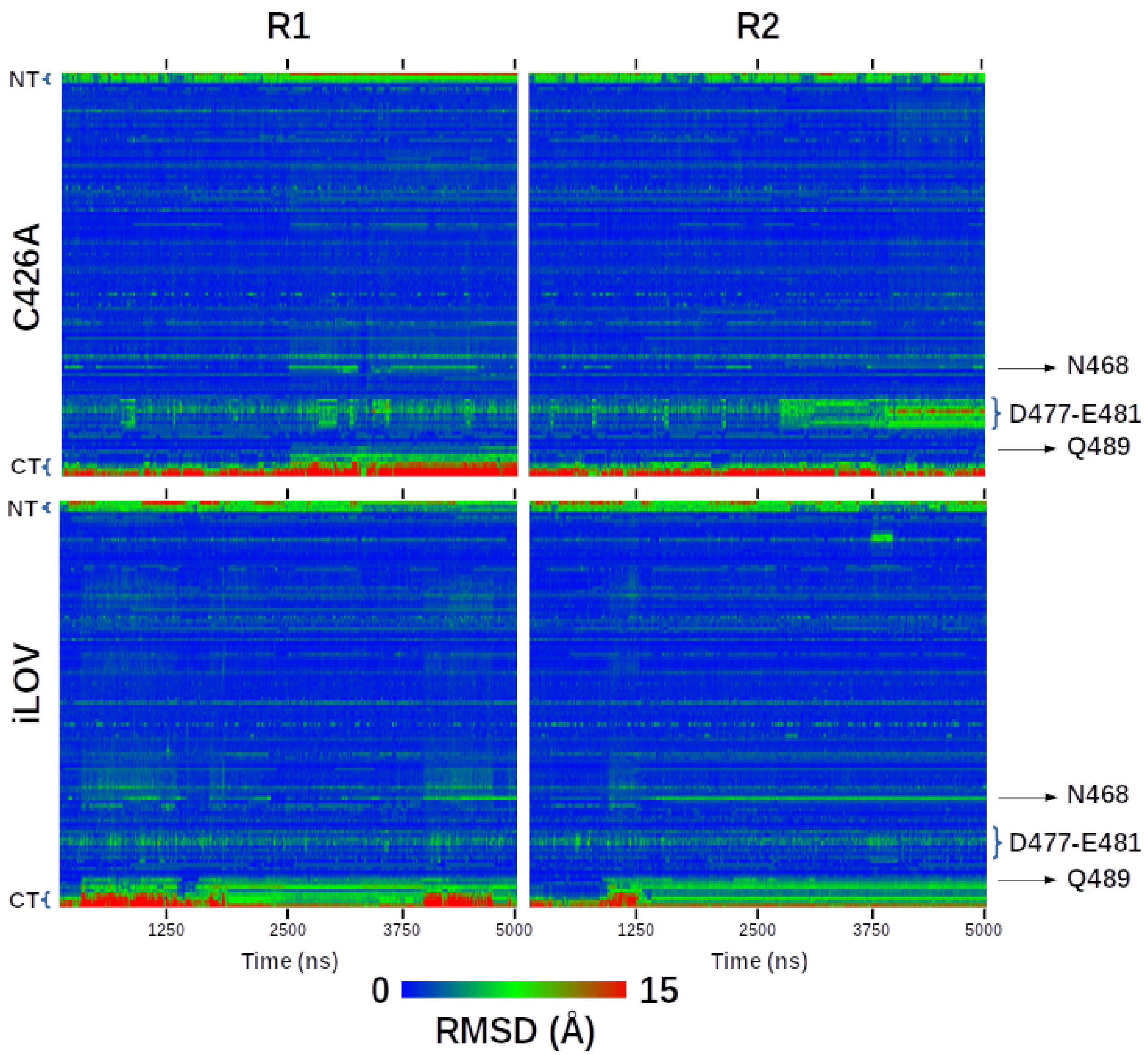

Figure S3: Per-residue RMSD calculated for C426A (top) and iLOV (bottom) employing both replicas R1 (left) and R2 (right). The crystal structures were used as reference for the fitting. Each plot shows the results for 5 μs of simulation (2500 frames were employed in this analysis). The arrows indicate the position of residues N468 (N82) and Q489 (Q103) whereas the parentheses show the position of the flexible loop between residues D477 and E481 (D91 and E95). NT and CT indicate, respectively, the N- and C-terminal ends. The regions in red have RMSD values equal to or greater than 15 Å.

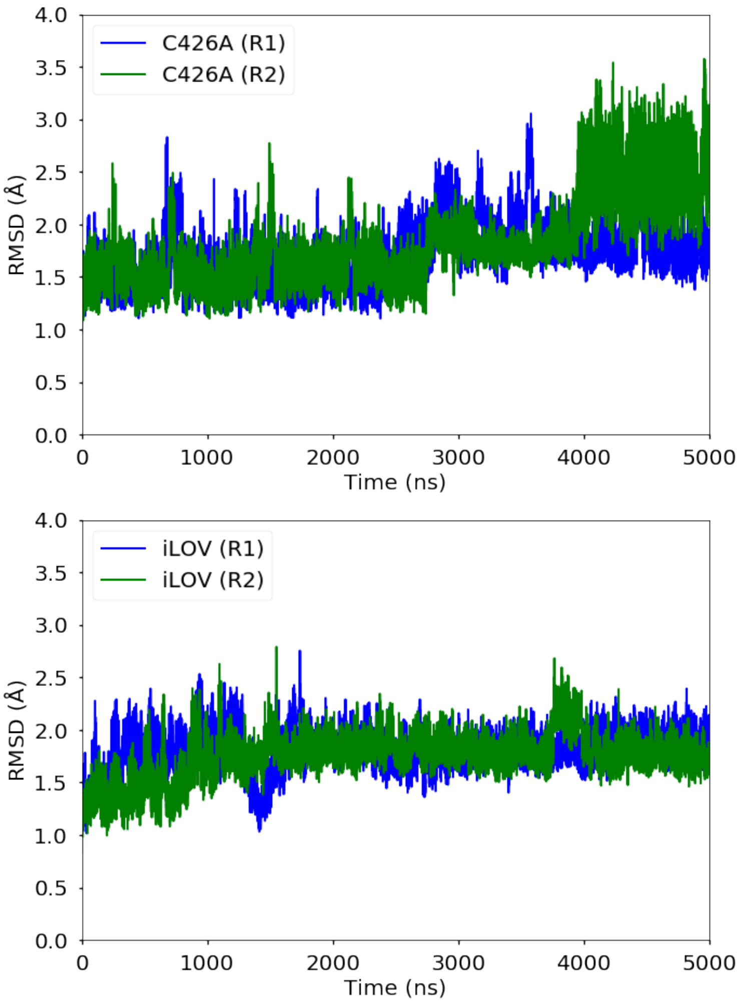

Figure S4: RMSD analysis of C426A (top) and iLOV (bottom) along each MD replica (R1 and R2). The N- and C-terminus were not considered for the structural fitting. The hydrogen atoms were excluded from the analysis.

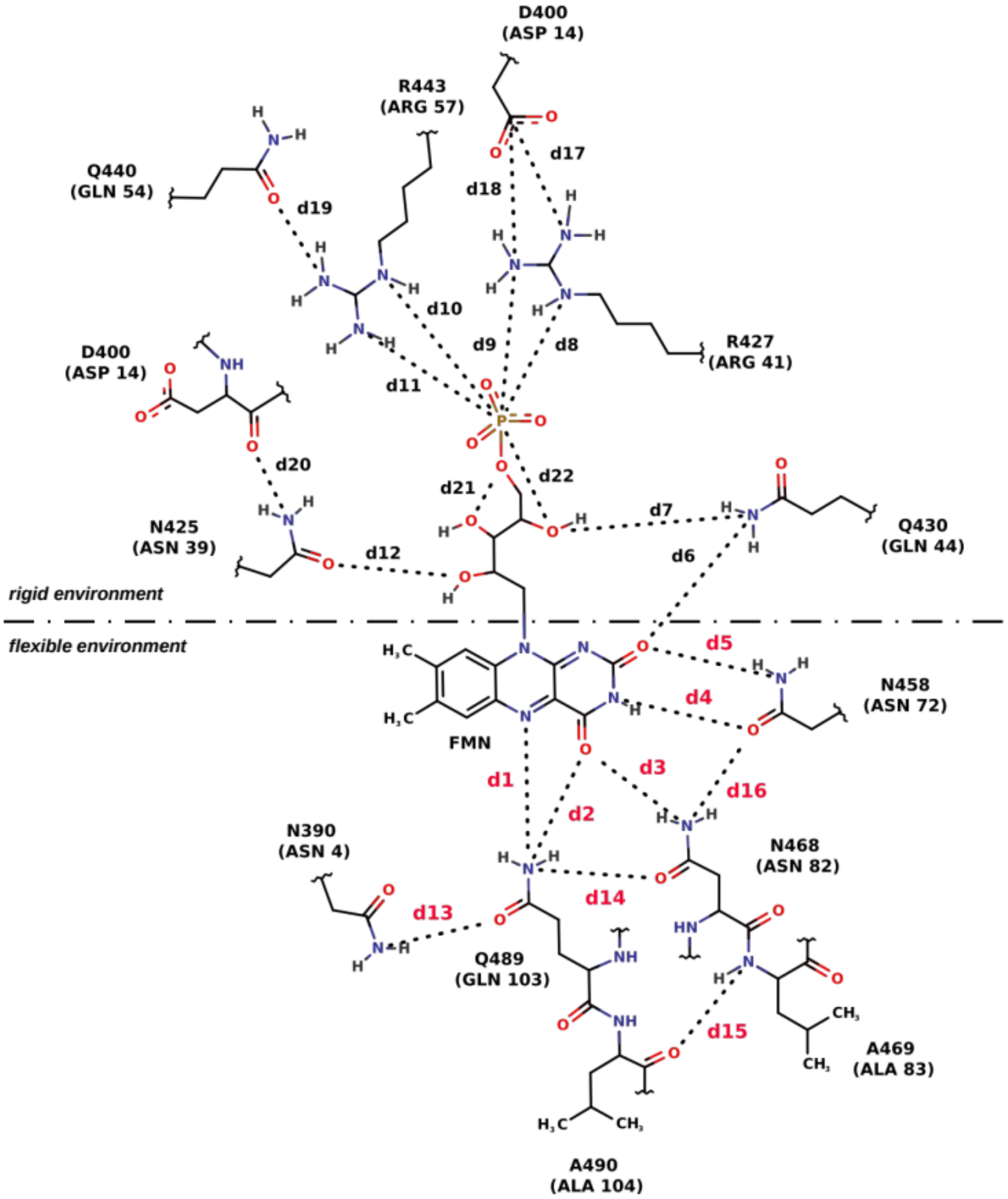

Figure S5: Map of interactions for some important H-bonds present in C426A and iLOV systems. We monitored 22 distances (d1-22) to study the stability of protein-FMN (d1-12), protein-protein (d12-20), as well as intra-FMN interactions (d21,22). Residue numbering is based on phot2 sequence, while the numbers in parentheses refer to the residue position in the synthetic protein sequences. This map can be divided into two parts: one part represents the rigid environment around the ribityl tail of FMN (upper part) and the other part represents the flexible environment of the FMN-binding site (lower part). In the rigid environment, the indicated H-bond showed to be quite persistent so that the network of interactions observed in the crystal structures were well reproduced in the MD simulations. The flexible environment, in turn, presented a complex H-bonding dynamics. Distances d1-5,d13-16, in red color, were employing for further analysis which resulted in obtaining of different clusters for C426A and iLOV, as discussed in the main text.

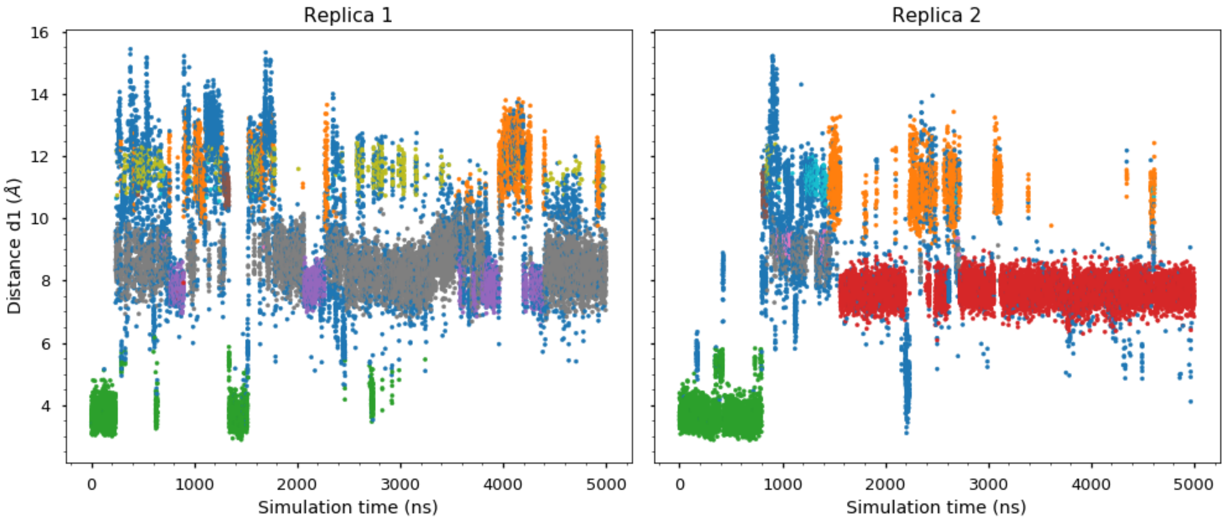

Figure S6: Distance d1 in the two iLOV replicas. The colors refer to the different clusters.

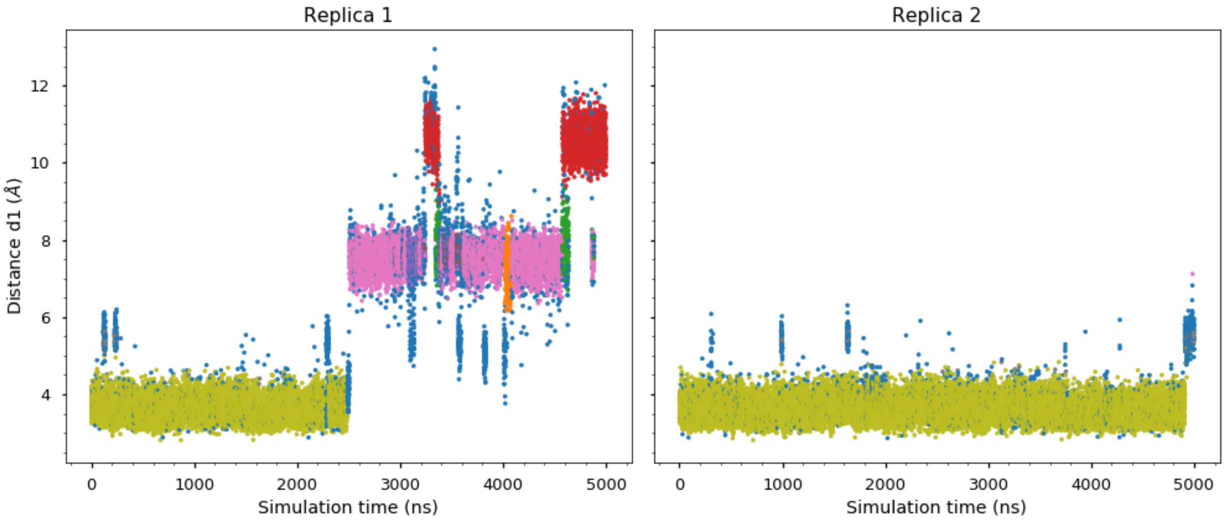

Figure S7: Distance d1 in the two C426A replicas. The colors refer to the different clusters.

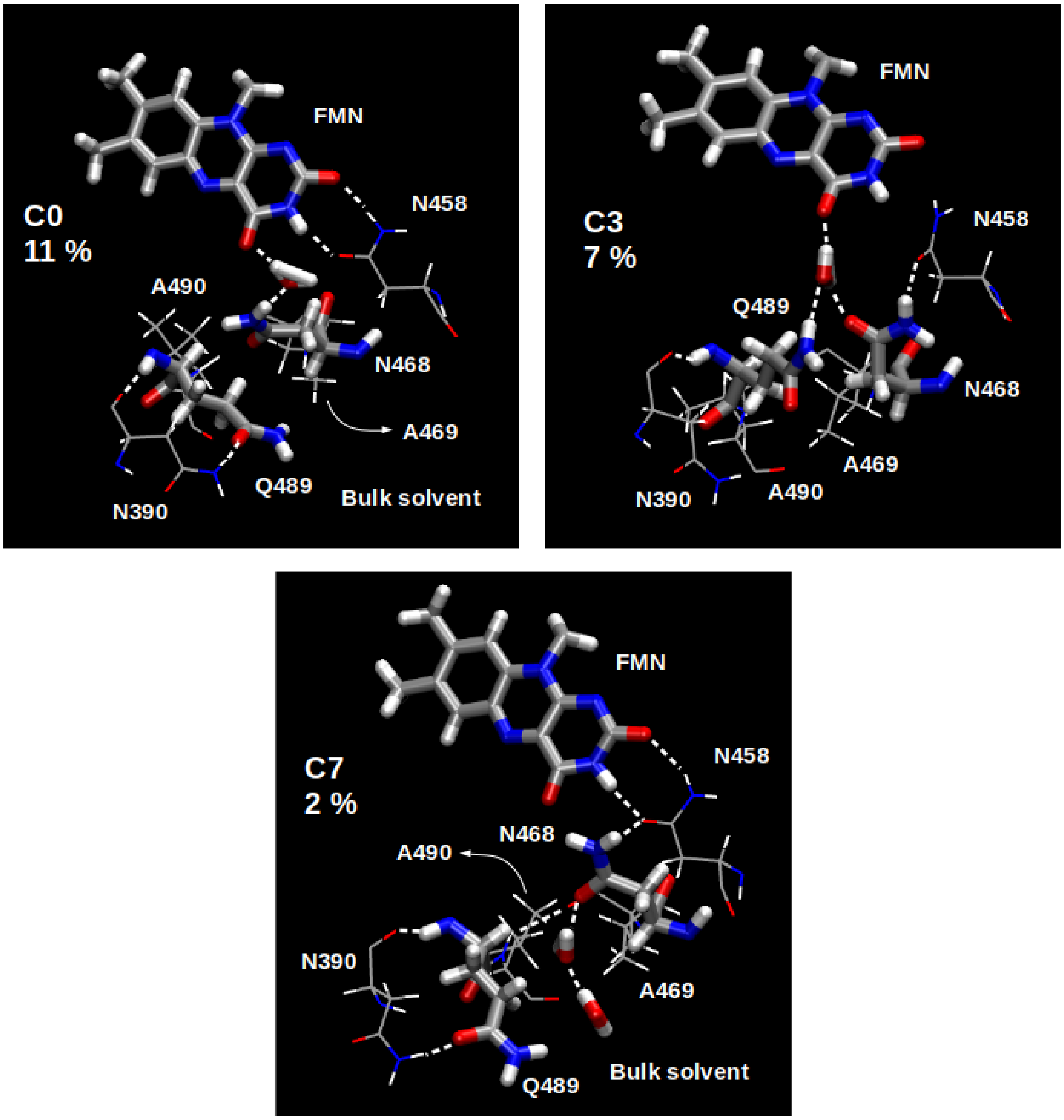

Figure S8: Less populated clusters obtained for iLOV (C0, C3 and C7).

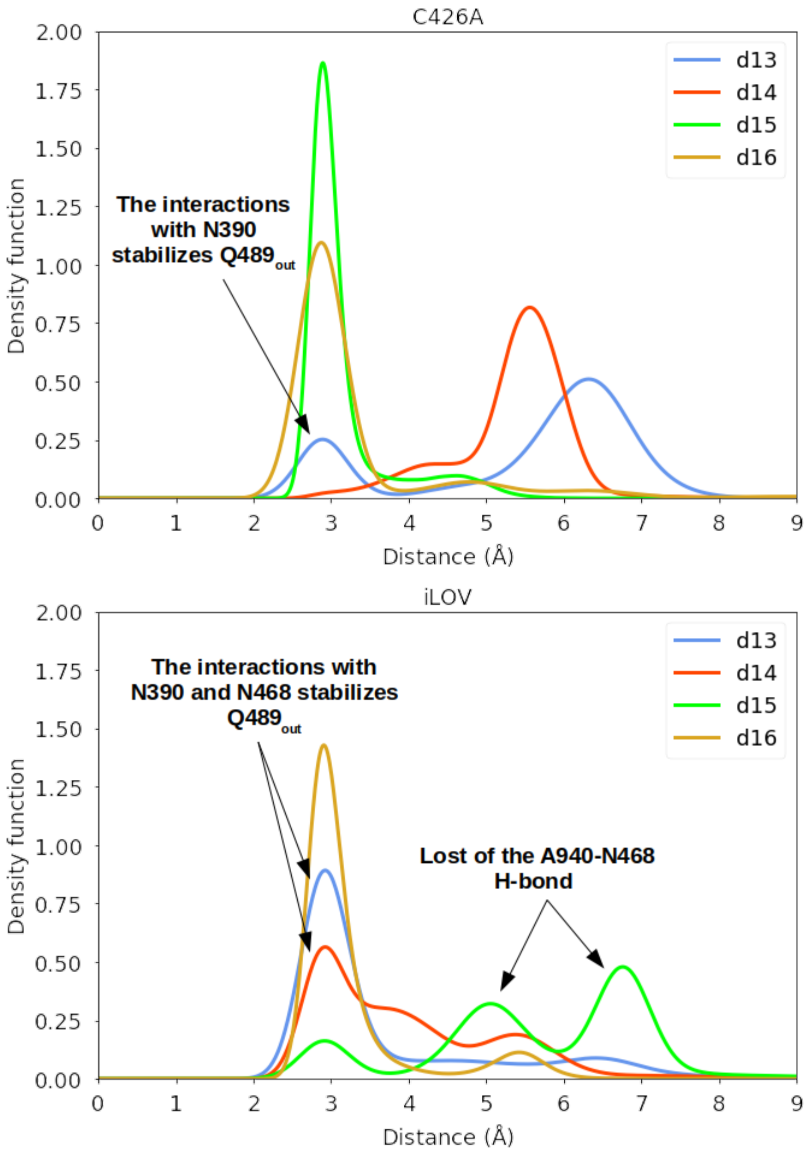

Figure S9: Distributions obtained for d13-15 distances in C426A (top) and iLOV (bottom).

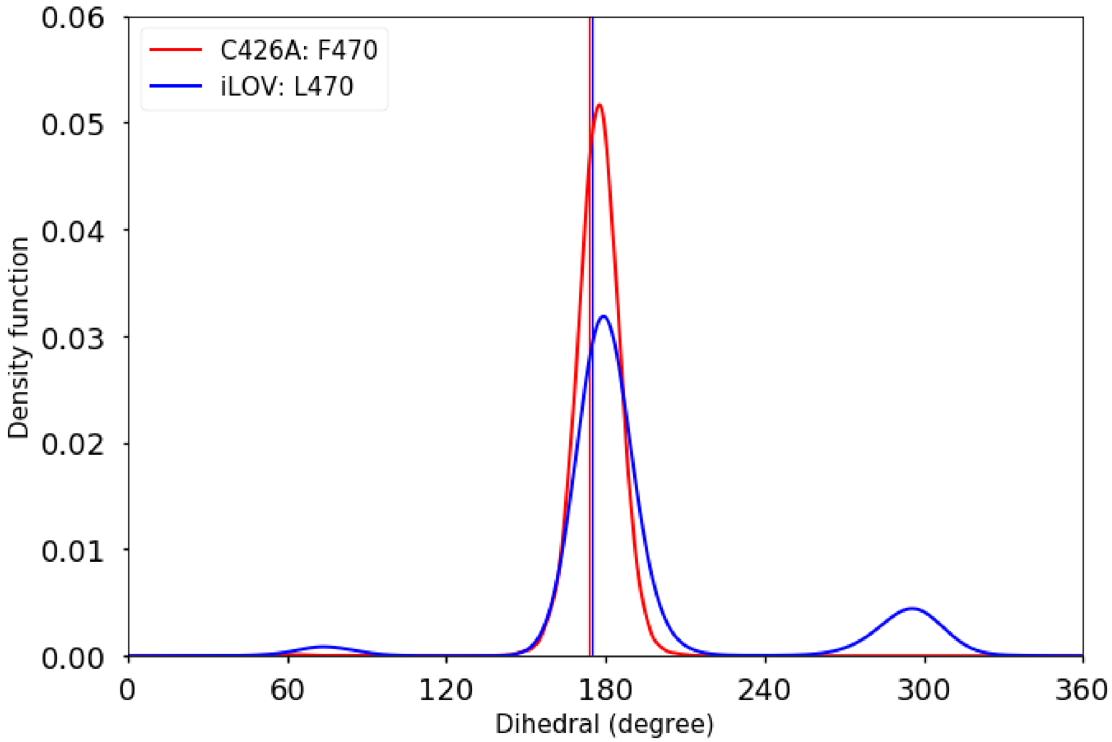

Figure S10: Dihedral distributions for F470 in C426A (red) and L470 in iLOV (blue). Vertical lines indicate the values obtained for crystal structures.

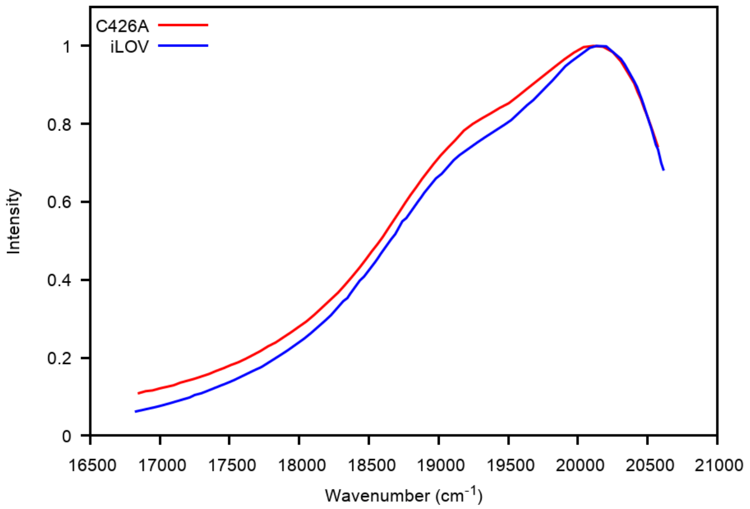

Figure S11: Comparison of the C426A and iLOV fluorescence spectra measured by Chapman *et al.*[1]

References

(1) Chapman, S.; Faulkner, C.; Kaiserli, E.; Garcia-Mata, C.; Savenkov, E. I.; Roberts, A. G.; Oparka, K. J.; Christie, J. M. The photoreversible fluorescent protein iLOV outperforms GFP as a reporter of plant virus infection. *Proc. Natl. Acad. Sci. U.S.A.* **2008**, *105*, 20038–20043.
